# Vacuum Packaging Maintains Fresh Characteristics of Previously Frozen Beef Steaks during Simulated Retail Display

**DOI:** 10.3390/foods11193012

**Published:** 2022-09-28

**Authors:** Madison P. Wagoner, Tristan M. Reyes, Virgina E. Zorn, Madison M. Coursen, Katie E. Corbitt, Barney S. Wilborn, Charles W. Starkey, Terry D. Brandebourg, Aeriel D. Belk, Tom Bonner, Jason T. Sawyer

**Affiliations:** 1 Department of Animal Sciences, Auburn University, Auburn, AL 36849, USA; 2 Department of Poultry Science, Auburn University, Auburn, AL 36849, USA; 3 Winpak Ltd., 100 Saulteaux Crescent, Winnipeg, MB R3J 3T3, Canada

**Keywords:** beef, fresh color, lipid oxidation, storage, slacked thaw, vacuum packaging

## Abstract

The impact of frozen storage on beef steaks prior to the retail setting may result in changes to the quality and safety of the packaged meat. Therefore, the objective of the current study was to evaluate fresh characteristics on previously frozen steaks during a simulated retail display. Steaks were allocated to one of three packaging treatments (MB, MDF, MFS) and stored frozen (−13 °C) for 25 days in the absence of light. After thawing, steaks were stored in a lighted retail case at 3 °C and evaluated for instrumental surface color, pH, purge loss, lipid oxidation, and microbial spoilage organisms throughout the 25-day fresh display period. There was an increase (*p* < 0.05) for aerobic plate counts and lipid oxidation from day 20 through 25 on steaks packaged in MFS and MDF, respectively. Steaks packaged in MB were redder (*p* < 0.05) and more vivid (C*) as storage time increased. Whereas lipid oxidation was greater (*p* < 0.05) throughout the entire display for steaks packaged in MFS and MDF. It is evident that barrier properties of MB limiting oxygen exposure of the steak preserved fresh meat characteristics after frozen storage. Results from the current study suggest that vacuum packaging films can aid in retarding detrimental effects caused by frozen storage after placing the steaks in fresh retail conditions.

## 1. Introduction

Meat products are highly perishable; therefore, strategies have been explored for countless decades to extend the fresh shelf life of red meat [1]. Cold storage is one such strategy frozen meat can extend storage life and the possible reduction in quality losses that occurs to fresh meat products [2,3]. Consumers may consider freezing meat purchases to prolong the interval between purchase and consumption. However, prior to consumer purchase the meat industry may consider frozen meat storage. If freezing meat prior to retail or foodservice use, this technique has often be used to extend storage periods, manage supply chain or facilitate distribution channels. However, despite the benefits, the process of frozen storage often requires a considerable amount of logistical planning that manufacturers or retailers may not find ideal.

During freezing, meat products undergo a physical transformation when water is converted into ice crystals, upon thawing, a transformation to a pre-frozen state occurs [4]. Freezing meat discourages food-borne pathogens by creating an unstable environment for microorganism growth, organism can regain activity as storage temperatures increase [5]. Despite the unavoidable physical changes, it is imperative the frozen-stored and thawed meat will retain the quality attributes consumers associate with fresh meat [6]. Moisture lost from the muscle during thawing can promote microbial growth as a as packaging purge increases [7]. Thus, the FDA and USDA-FSIS have indicated to consumers meat should be thawed using refrigerated temperature at 4.44 °C or below to discourage microbial growth [8,9]. In addition to microbial growth, other important meat characteristics that are affected by freezing and thawing procedures also include: moisture loss, protein denaturation, lipid oxidation, surface color, pH, objective tenderness, and purge loss [10,11].

At the point of sale, the visual appearance of beef products represents the most important characteristic influencing consumer purchasing decisions with a characteristic cherry-red color being highly desirable [12,13]. Vacuum packaging limits meat surface exposure to oxygen resulting a dark purple. However, when permeability of the packaging film increases, greater concentrations of oxygen can all the meat surface to possess a bright, cherry-red color.

Often, the most overlooked packaging factor to consider when selecting packaging materials for meat products is the oxygen transmission rate (OTR) of the packaging film. Packaging film OTR reflects the potential for oxygen and other atmospheric gases to bind with myoglobin and form surface color pigments, thus there may be an optimal OTR which would promote the reddish surface color that consumers prefer at the time of purchase [14]. Additionally, moisture vapor barrier properties may also influence storage of fresh and frozen meat [15]. Although freezing meat offers consumers a product that reflects the same nutritional quality as fresh products, physical and biochemical changes that occur to meat during freeze storage can negatively affect critical organoleptic properties like surface color [14].

Purge loss is inevitable in fresh meat given the inherent conversion of muscle to meat driving such processes as rigor mortis and postmortem muscle pH [15]. Retaining moisture in meat products is important to limit the loss of salable weight and protein at the time of consumer purchase [16]. Furthermore, there is a mechanistic relationship between pH and water holding capacity as the loss of hydrogen ions (H^+^) can accelerate pH decline and reduced water-holding capacity can lead to unacceptable purge loss [7,16]. Increased thawing time at elevated temperatures may also increase purge loss promoting an increase in microbial proliferation [17]. To date, limited research investigating the impact of packaging methods and materials on meat quality has been published [18]. Therefore, the objective for the current study was to determine the effect of vacuum packaging on shelf-life characteristics of boneless ribeye steaks that have been previously frozen.

## 2. Materials and Methods

### 2.1. Muscle Fabrication

Beef boneless ribeye rolls (IMPS #122A) were purchased from a commercial meat processor and transported under refrigeration (2 °C) to the Auburn University Lambert Powell Meat Laboratory for processing. Using pack date on each box not exceeding 10 days from the time of packaging, ribeye rolls were selected for steak cutting. Ribeye rolls (N = 18) were fabricated into 2.54-cm-thick steaks (n = 12 steaks/ribeye roll) with a BIRO bandsaw (Model 334, Biro Manufacturing Company, Marblehead, OH, USA). Steaks from each ribeye roll were randomly selected and allocated to one of three packaging treatments.

### 2.2. Packaging Treatments

After cutting, steaks were allowed to bloom to simulate an industry application for 30 min at 2 °C, crust frozen at −23 °C for 45 min, and then packaged with a form and fill packaging machine (Model OL0924, Variovac, Zarrentin, Germany). Steaks were packaged in one of three commercially available packaging films (WINPAK, Winnipeg, MB, Canada) consisting of a high barrier and or low barrier film. The high barrier film (MB) was comprised of 150 μm of nylon, enhanced ethylene-vinyl alcohol (EVOH), and polyethylene. Steaks packaged in low barrier films were constructed with 150 μm polypropylene and polyolefin plastomer (MFS) or a combination of 150 μm polyolefin and polyethylene (MDF). Oxygen transmission (OTR) of the packaging treatmentss consisted of: MB (0.5 cc/sq. m/24 h); MFS (1100 cc/sq. m/24 h); and MDF (1287 cc/sq. m/24 h). In addition, moisture vapor transmission of each packaging film was measured: MB (3.9 g/sq. m/24 h); MFS (2.9 g/sq. m/24 h); and MDF (3.5 g/sq. m/24 h). Packaged steaks were placed flat on a tray (76.2 cm × 60.96 cm) and stored in a blast freezer (−23 °C) for 120 min.

### 2.3. Simulated Storage Periods

Initially, steaks were placed in a two-door, reach-in, commercial freezer (Model AF49EX, Arctic Air, Eden Prairie, MN, USA) for 25 days at −13 °C. Packaged steaks were stored in the absence of light for the duration of the simulated frozen storage period. Temperature during the frozen storage period was monitored using a data recording device (Model-TD2F, Thermoworks, American Fork, UT, USA) with probes placed within the center of each shelf. Throughout the storage period frozen steaks were rotated across all shelves.

Following the 25-day frozen dark storage, packaged steaks were transferred to an LED lighted, refrigerated, 3-tiered, case (Model TOM-60DX-BN, Turbo Air Inc., Long Beach, CA, USA) to simulate a fresh retail setting. Packaged steaks were displayed at 3 °C ± 1.2 °C and data loggers (Model- TD2F, Thermoworks, American Fork, UT, USA) recorded storage temperatures. Continuous lighting intensity (2297 lux) of case shelves was recorded (Model ILT10C, International Light Technologies, Peabody, MA, USA) throughout the fresh display period. During fresh display, steaks were placed across all shelves and rotated on the shelving to simulate consumer movement. On days 0, 7, 10, 15, 20, and 25 steaks were removed from the refrigerated display case and measured for instrumental color, lipid oxidation, purge loss, pH, and spoilage organisms.

### 2.4. Instrumental Color

Fresh instrumental color readings were measured through the packaing on day 0, 5, 10, 15, 20, and 25 by scanning the surface of each steak through the packaging according to guidelines previously described [19]. Surface color values were collected using a HunterLab MiniScan XE Plus Colorimeter (Model 45/0-L, Hunter Associates Laboratory Inc., Reston, VA, USA) calibrated against a standard black and white glass tile each day immediately before data collection. The L* (lightness), a* (redness), and b* (yellowness) values of each steak were determined from the average of three readings using Illuminant A10, with a 10° observer and a 25 mm diameter aperture and the Commission Internationale de l’ Eclariage (CIE L*a*b*) color scale [20]. Chroma (C*) was calculated using the following equation: √a*^2^ + b*^2^ with a more vivid color resulting from a great value. Additionally, hue angle was calculated as: tan^−1^ (b*/a*) where a greater value represents the surface color shifting from red to yellow.

### 2.5. Microbial Analysis: Aerobic Plate Count

The total number of viable non-pathogenic aerobic microorganisms was determined using standard methods [21]. Duplicate 5 g samples were removed aseptically from each package. Samples were placed in a stomacher bag containing a sterile filter (3M Corp., St. Paul, MN, USA) and 50 mL of Butterfield’s Buffer (3M Corp., St. Paul, MN, USA). Stomacher bags were agitated for 1 min. After stomaching, a 10-fold dilution series was completed for microbial analysis. Serial, duplicate platings were placed onto aerobic (APC) plates, Petrifilm^®^ (3M Corp., St. Paul, MN, USA), and incubated at 36.0 °C for 48 hours in an incubator chamber (Model IB-05G, Lab Companion, Yuseong-gu, Daejeon, Korea) prior to enumeration. Microbial counts were recorded as colony-forming units per gram (CFU/g) [21]. Incubation temperature was recorded using a data logger (Model-TD2F, Thermoworks, American Fork, UT, USA) placed in the geometric center of each self.

### 2.6. Lipid Oxidation

Packages of fresh steaks were sampled for 2-thiobarbituric acid reactive substances (TBARS) as previously described [22]. Steaks were minced into a uniform sample of the entire steak with a hand-held knife. In duplicate, 2 g ± 0.5 g of each minced steak was pulverized with 8 mL of cold (1 °C) of 50 mM phosphate buffer (pH of 7.0 at 4 °C) containing 0.1% ethylenediaminetetraacetic acid (EDTA), 0.1% n-propyl gallate, and 2 mL trichloroacetic acid (Sigma-Aldrich, Saint Louis, MO, USA). Samples were filtered through a Whatmann No. 4 filter paper and duplicate 2-mL aliquots of the clear filtrate were transferred into 10-mL borosilicate tubes, mixed with 2 mL of 0.02 M 2-thiobarbituric acid reagent (BeanTown Chemical, Hudson, NH, USA) and boiled at 100 °C for 20 min. After boiling, tubes were placed into an ice bath for 15 min. Absorbance of each sample was measured at 533 nm with a spectrophotometer (Turner Model-SM110245, Barnstead International, Dubugue, IA, USA) and multiplied using a factor of 12.21 to derive the TBARS value (mg of malonaldehyde/kg of fresh meat). The value of 12.21 was obtained previously from a standard curve using a known malonaldehyde solution measured across multiple absorbencies [22].

### 2.7. Fresh pH

Muscle pH was measured on steaks throughout the fresh display period in duplicate using a steel electrode attached to a pH meter (Model HI199163, Hanna Instruments, Woonsocket, RI, USA) inserted into the steak at two random locations. Prior to collecting fresh muscle pH values, the pH meter was calibrated using 2-point (4.0 and 7.0) buffers (Thermo Fisher Scientific, Chelmsford, MA, USA) and after every 5 readings.

### 2.8. Purge Loss

Steaks were removed from their package treatment, blotted dry with a paper towel and weighed on a balance (Model PB3002-S, Mettler Toledo, Columbus, OH, USA). Purge loss calculations is as follows: [(packaged weight − steak weight) ÷ packaged weight × 100)].

### 2.9. Statistical Analysis

The current study was conducted and analyzed as a completely randomized design with packaged steaks serving as the experimental unit. Data were analyzed using the GLIMMIX model procedure of SAS (version, 9.2; SAS Inst. Inc., Cary, NC, USA). Packaging treatment was used as a lone fixed effect and replication represented the random effect for instrumental surface color, APC, TBARS, pH, and purge loss. Day of simulated display served as a repeated measure, whereas packaging treatment, day, and packaging treatment × day interaction were fixed effects. Least square means were generated, and when significant (*p* ≤ 0.05) F-values observed, least square means separation occurred by using the pair-wise t-test (PDIFF option).

## 3. Results and Discussion

### 3.1. Instrumental Color

Following frozen dark storage, instrumental color of vacuum-packaged steaks was recorded throughout a 25-day fresh display period. There was no interaction (*p* > 0.05) for packaging method × day of display for fresh surface color lightness (L*; values not reported). However, throughout the fresh display there was an interaction (*p* < 0.05) for packaging method × day of simulated display for redness (a*), yellowness (b*), chroma (C*) and hue angle (Table 1). Steaks packaged using MB film were redder (*p* < 0.05) from day 7 through 25 of the fresh simulated retail display period. However, steaks packaged in MFS and MDF films were more yellow (*p* < 0.05) initially, but as storage time increased past day 20, yellowness values declined for all packaging methods. This data suggest that the use of MB film may promote a better visual color during retail display post frozen storage compared to MFS and MDF films.

A decline in a* values of thawed meat has been attributed to myoglobin denaturation occurring during colder storage temperatures, but surface redness can increase after thawing when myoglobin is stored in a favorable oxygen binding environment [23]. A similar study conducted examining the relationship between frozen and fresh beef color values reported increased anaerobic refrigerated storage duration can result in a rapid decline of a* values which has also been linked to an increase in lipid oxidation [24]. Our results are consistent with a previous study that evaluated the surface color of meat following a frozen storage period and reported declining b* values throughout a refrigerated storage time after frozen storage [25]. However, another study reported b* values did not differ after thawing at different temperatures when measuring the surface color of beef *Longissimus dorsi* [26]. Interestingly, duration of storage time may negatively influence the percentage of oxymyoglobin or metmyoglobin causing a detrimental impact on redness values for steaks possessing greater percentages of oxymyoglobin [27]. Nevertheless, it has been reported that increasing oxygen saturation prior to freezing can result in greater oxidation after thawing, and a loss of reducing enzymes through exudate contributing to a deterioration in color stability [13,27]. Storage temperature of frozen black wildebeest resulted in yellowness (b*) values increasing during the refrigerated storage time, but these surface color changes of game meats may have occurred because of inherent darker color and greater muscle pH values related to game muscle [28]. Results from the current study support the hypothesis that surface color may be negatively altered after storage in frozen and subsequent refrigerated temperatures.

Consistent with observed redness and yellowness values for thawed beef steaks, instrumental surface color was also more (*p* < 0.05) vivid (C*) for steaks packaged in MB from day 7 through day 25 of the fresh display period (Table 1). In contrast, hue angle values for MFS and MDF increased (*p* < 0.05) throughout the entire display period indicative of color shifting from red to yellow for these packaged steaks. The current results for C* differ from previous results that have reported the combination of chilled-then-freezing beef loins can cause an increase in C* values with increased storage time [29]. However, additional studies have reported that storing frozen beef in oxygen impermeable films resulted in maintenance of a more desirable color, reduced off-flavors and less lipid oxidation [30,31]. In the current study, packaging steaks using MB film constructed with the lowest OTR rating appeared to confer protection against deterioration throughout the storage periods to thawed beef steaks resulting in greater surface color stability. It has been reported that color changes throughout frozen storage in marinated raw beef meat can be due myoglobin denaturation caused by lipid oxidation [32]. Additionally, it has been reported that a decrease in lightness values during the freeze–thaw cycle is associated with a surface light reflectance attributable to water loss [32]. Color shifting in frozen meat may be caused by physical processes such as drip loss, or when water molecules freeze resulting in a shift of fat, total protein, and water/protein ratio chemical concentrations on the surface layer after thawing [33]. It is plausible that the duration of frozen storage time, packaging materials, and refrigerated storage temperature will affect color stability. Further research is needed determine the impact of intrinsic and extrinsic factors on meat color stability when storage temperatures are altered. Regardless, these data point to a potential advantage for MB packaging film.

### 3.2. Aerobic Plate Count Changes

An interaction (*p* < 0.05) for packaging treatment × day of retail display occurred for spoilage organisms (Table 2). Regardless of packaging treatment, spoilage organisms increased (*p* < 0.05) throughout the simulated refrigerated display period. However, spoilage organism growth was hindered (*p* < 0.05) when using MB for packaging steaks after day 20. Interestingly, it should be noted that throughout the duration of the current study, there were no packaging treatments that crossed the 6 log CFU/g threshold thus no packing treatments associated with detrimental effects on the wholesomeness and safety of fresh meats in the current study.

Results agree with previous findings related to varying storage treatments (ex. chilled only, frozen only, or chilled then frozen) where like in the current study, the storage of beef loins did not cause an increase in spoilage microorganisms [29]. Furthermore, reduced the growth of microbial populations has been reported for meat when stored at colder temperatures (−12 °C to −18 °C) [29]. Nonetheless, an increased microbial count is to be expected after freezing and thawing because of exudate formation coupled with an increase in moisture and the amount of nutrients available to support microbial proliferation [7]. Packaging materials that are constructed to limit OTR can reduce oxygen transmission thus reducing aerobic microbial proliferation and potentially extend shelf life. Limited microbial growth in the current study agrees with a previous study that examined packaging OTR and the subsequent influence on aerobic spoilage organisms [34].

### 3.3. Lipid Oxidation

**Figure 1:**
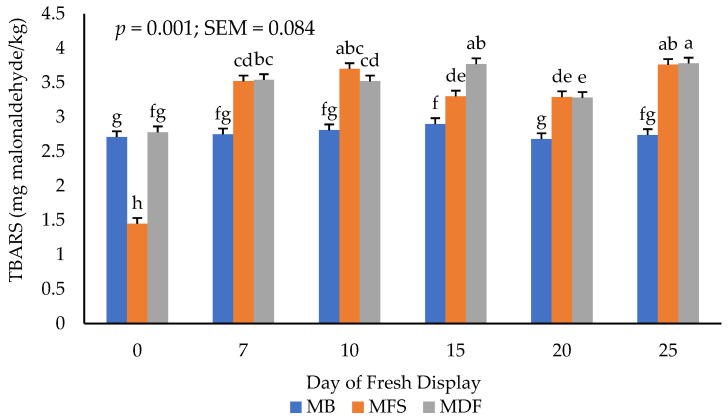
Interactive effect of treatment × day of display for 2-Thiobarbituric acid reactive substances (TBARS). a–h: Bars lacking common letters differ (*p* ≤ 0.05).

There was an interactive effect of packaging method × day of display for lipid oxidation on thawed beef steaks (Figure 1). Lipid oxidation increased (*p* < 0.05) in steaks packaged using MDF and MFS films throughout the 25-day simulated retail display. Increases in lipid oxidation values agree with previous findings that evaluated vacuum stored meat products. The reduced lipid oxidation reported in MB-versus MDF, and MFS films is expected as a reduced OTR would reduce exposure of the steak to oxidation throughout the frozen and fresh storage periods. Consistent with this hypothesis, an accelerated rate of lipid oxidation associating with a greater amount of oxygen exposure has been reported across packaging materials [24,25,26,28,29,32,33,35]. In a study examining minced porcine muscles stored in vacuum packaging, lipid oxidation tended to accelerate after thawing as peroxidation giving rise to rapid secondary lipid oxidation and increased TBARS values were reported [36]. In the current study, it appears the MB film confers a greater protection against lipid oxidation than the use of either MDF or MFS films.

### 3.4. Fresh pH

**Figure 2:**
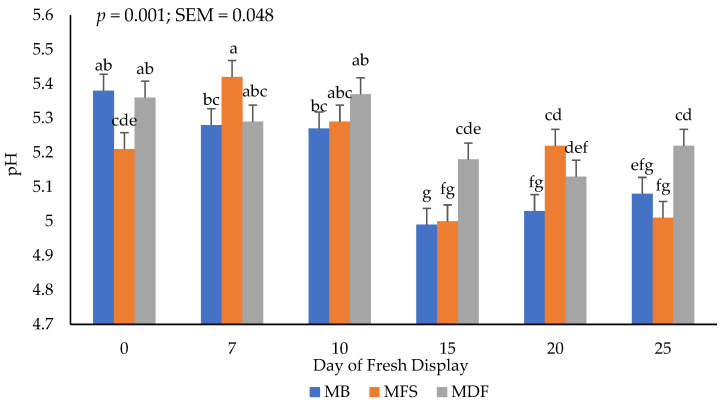
Interactive effect of packaging method × day of display on fresh pH values. a–g: Bars lacking common letters differ (*p* ≤ 0.05).

The interactive (*p* < 0.05) of packaging method × day of display for pH values of thawed steaks is presented in Figure 2. Postmortem muscle pH can be instrumental in indicating the quality of fresh meat surface color and optimal pH values may hinder microbial growth. In the current study, a significant decrease (*p* < 0.05) in pH occurred across packaging treatments throughout the stimulated storage periods. It is worth noting that in this study, fresh muscle pH values did not decline below values that would be expected to detrimentally influence surface color values.

It has been previously stated that greater pH values have been attributed to the denaturation of buffer proteins with the increase of solute concentration occurring in frozen storage [37]. It is plausible that an increase of OTR for steaks packaged in MDF influenced the growth in lactic acid bacteria often noted in vacuum-packaged meats [38]. A similar reduction in muscle pH was reported when evaluating frozen vacuum packaged meat after frozen storage [25]. Moreover, the loss of free moisture from the meat products during the defrosting phase can result in a greater concentration of solutes within the package, plausible causing a decline in pH of thawed meat [37]. Conversely, packaging methods of frozen beef sirloins did not appear to influence muscle pH [11]. However, lactic acid bacteria are generally associated with a decline in muscle pH (<5.8) when packaging meats under vacuum due to a reduced oxygen environment [39,40]. Previous studies do not agree with our current results whereby packaging method may influence postmortem muscle pH, although the literature addressing this topic is limited focused on frozen then thawed meat pH.

### 3.5. Purge Loss

**Figure 3:**
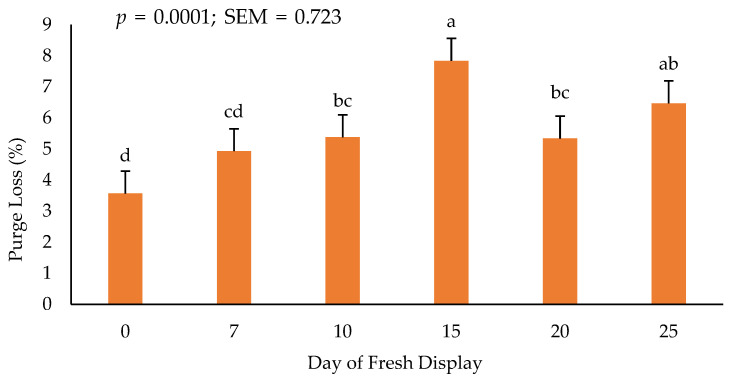
Influence of display day on purge loss (%) of refrigerated ribeye steaks. a–d: Bars lacking common letters differ (*p* ≤ 0.05).

There was no interactive effect for packaging treatment × day on purge loss of packaged steaks (values not reported). Purge loss after frozen storage and throughout fresh, refrigerated storage increased (*p* < 0.05) from day 0 to 25 regardless of packaging (Figure 3). It is plausible that the rise in purge loss could be attributed to the decline in muscle pH that occurred across all packaging treatments causing greater amounts of free and bound water to be lost during storage.

Purge loss, specifically water holding capacity, is related to the available moisture properties residing within microfibrillar proteins [11]. Furthermore, moisture losses occurring in meat have been linked to storage temperatures and temperature variation can influence moisture loss during storage periods [40]. Given this, purge loss remains a crucial factor to consider when selecting packaging materials or storage temperatures due to the monetary impact throughout the meat industry even though we observed no differences in purge loss related to packaging film for previously frozen, beef ribeye steaks across MB, MSF, and MDF films.

## 4. Conclusions

Results presented here supports the hypothesis that when selecting vacuum-packaging film during the storage of beef products, oxygen transmission and moisture vapor transmission rate of the film should be considered. The potential influence caused by packaging film composition can alter surface color, and wholesome characteristics throughout a freeze–thaw cycle of beef steaks. It is plausible that steaks packaged in film possessing reduced oxygen and moisture transmission rates may have a more stable surface color, reduced lipid oxidation, and hindered aerobic microorganism growth. However, to enhance the consumer acceptance of vacuum packaging, additional educational opportunities should be provided to consumers and producers on the various impacts of freezing and thawing of vacuum packaged red meats. Furthermore, evaluating the sensory profile of meat products after freeze–thaw cycles when using vacuum packaging films for red meat storage is needed.

## Figures and Tables

**Table 1:** Interactive impact of packaging method × day of display on instrumental color values.

	Day						
	0	7	10	15	20	25	SEM *
**MB ^1^**							
a* ^2^	13.61 ^e^	22.11 ^a^	20.45 ^b^	20.15 ^b^	17.52 ^c^	15.76 ^d^	0.330
b* ^2^	13.94 ^fg^	13.17 ^hi^	13.03 ^hi^	13.77 ^gh^	13.59 ^ghi^	12.88 ^hi^	0.251
C* ^3^	19.69 ^e^	25.79 ^a^	24.27 ^b^	24.45 ^b^	22.25 ^d^	20.40 ^e^	0.326
Hue (°) ^4^	45.87 ^d^	30.85 ^i^	32.52 ^h^	34.41 ^g^	38.03 ^f^	39.51 ^f^	0.716
**MFS ^1^**							
a* ^2^	17.21 ^c^	10.46 ^gh^	10.46 ^gh^	10.78 ^fgh^	10.03 ^hi^	10.17 ^ghi^	0.345
b* ^2^	16.28 ^a^	16.25 ^ab^	15.33 ^cd^	14.66 ^ef^	13.94 ^fg^	13.92 ^fgh^	0.251
C* ^3^	23.76 ^bc^	19.94 ^e^	18.61 ^f^	18.32 ^fg^	17.25 ^h^	17.29 ^gh^	0.344
Hue (°) ^4^	43.63 ^e^	54.83 ^b^	55.85 ^b^	53.90 ^b^	54.36 ^b^	53.69 ^b^	0.716
**MDF ^1^**							
a* ^2^	17.04 ^c^	11.25 ^fg^	9.19 ^i^	10.29 ^gh^	10.69 ^fgh^	11.98 ^f^	0.345
b* ^2^	15.79 ^bc^	16.05 ^ab^	14.99 ^de^	14.34 ^fg^	12.99 ^hi^	12.75 ^i^	0.251
C* ^3^	23.31 ^c^	19.65 ^e^	17.62 ^fgh^	17.72 ^fgh^	16.94 ^h^	17.56 ^fgh^	0.344
Hue (°) ^4^	43.06 ^e^	55.24 ^b^	58.58 ^a^	54.34 ^b^	50.45 ^c^	47.06 ^d^	0.716

^1^ Packaging treatments: (MB) nylon + enhanced ethylene-vinyl alcohol + polyethylene; (MFS) polypropylene + polyolefin plastomer; and (MDF) polyolefin + polyethylene. ^2^ a* values measure redness (larger value indicates a redder color); and b* values measure yellowness (larger value indicates a more yellow color). ^3^ Chroma measures total color (larger number indicates a more vivid color). ^4^ Hue angle is the change from the true red axis (larger number indicates a greater shift from red to yellow). * SEM, standard error of the mean. ^a–i^ Mean values within day of display and packaging method lacking common superscripts differ (*p* < 0.05).

**Table 2:** Interactive effect of packaging method × day on aerobic (APC) spoilage organism growth.

	Day					SEM *
	0	10	15	20	25	
**MB ^1^**	>0.001 ^e^	0.57 ^d^	1.25 ^ab^	0.85 ^bcd^	0.57 ^d^	0.142
**MFS ^1^**	0.04 ^e^	0.56 ^d^	0.86 ^bcd^	1.46 ^a^	1.03 ^bc^	0.142
**MDF ^1^**	>0.001 ^e^	0.83 ^cd^	0.85 ^cd^	1.47 ^a^	1.44 ^a^	0.142

^1^ Packaging treatments: (MB) nylon + enhanced ethylene-vinyl alcohol + polyethylene; (MFS) polypropylene + polyolefin plastomer; and (MDF) polyolefin + polyethylene. * SEM, standard error of mean. ^a–e^ Mean values within day of display and packaging treatment lacking common superscripts differ (*p* < 0.05).

## Data Availability

The data presented in this study are available on request from the corresponding author.
